# Systematic Review on Upper Urinary Tract Carcinoma in Kidney Transplant Recipients

**DOI:** 10.3390/jcm14113927

**Published:** 2025-06-03

**Authors:** Alberto Piana, Alicia López-Abad, Battista Lanzillotta, Alessio Pecoraro, Thomas Prudhomme, Hakan Bahadır Haberal, Michele Di Dio, Beatriz Bañuelos Marco, Muhammet Irfan Dönmez, Alberto Breda, Angelo Territo

**Affiliations:** 1 Department of Urology AOU San Luigi Gonzaga, University of Turin, 10043 Orbassano, Italy; 2 Department of Urology, Virgen de la Arrixaca University Hospital, 30120 Murcia, Spain; alicialopezabad@gmail.com; 3 Division of Urology, Department of Surgery, Annunziata Hospital, 87100 Cosenza, Italy; battistalanz@gmail.com (B.L.); m.didio@aocs.it (M.D.D.); 4 Department of Experimental and Clinical Medicine, University of Florence, 50134 Florence, Italy; alessiopecoraro10@gmail.com; 5 Department of Urology, Kidney Transplantation and Andrology, Toulouse Rangueil University Hospital, 31400 Toulouse, France; prudhomme.t@chu-toulouse.fr; 6 Department of Urology, Ankara Ataturk Sanatorium Training and Research Hospital, Ministry of Health, University of Health Sciences, 06290 Ankara, Turkey; bahadirhaberal@gmail.com; 7 Division Renal Transplantation and Reconstructive Urology, Hospital Universitario El Clínico San Carlos, 28040 Madrid, Spain; banuelosmarco@gmail.com; 8 Department of Urology, Istanbul Faculty of Medicine, Istanbul University, 34093 Istanbul, Turkey; m_irfan83@yahoo.com; 9 Unit of Uro-oncology and Kidney Transplant, Department of Urology, Puigvert Foundation, Universitat Autònoma de Barcelona (UAB), 08025 Barcelona, Spain; albbred@gmail.com (A.B.); territoangelo86@gmail.com (A.T.)

**Keywords:** upper urinary tract urothelial cell carcinoma, kidney transplantation, nephroureterectomy, recipients

## Abstract

**Objectives:** Upper urinary tract urothelial cell carcinoma (UTUC) in kidney transplant recipients (KTRs) presents distinct clinical challenges due to the complexities of managing both cancer and the long-term immunosuppressive therapy required to preserve graft function. UTUC in this population often presents at advanced stages, contributing to poorer outcomes compared to immunocompetent individuals. **Methods:** This systematic review (SR) evaluates the incidence, clinical presentation, treatment approaches, and survival outcomes of UTUC in KTRs, based on 16 retrospective studies including 526 patients. **Results:** The present study highlights a predominance of female patients (ranging from 50% to 91.6%) and significant variability in time to diagnosis (from 7 to 181 months post-transplant). Tumor characteristics also showed considerable heterogeneity, with high-grade and advanced-stage (T3–T4) tumors being more common. The standard treatment for UTUC in KTRs remains radical nephroureterectomy (RNU), with additional resection of the bladder (TURB) when bladder cancer (BC) coexists. Survival outcomes vary significantly, with 5-year overall survival (OS) rates ranging from 16.7% to 90.9%, strongly influenced by tumor stage at diagnosis. This SR further reports high rates of bladder recurrence (18.8% to 61.2%) and challenges in balancing effective cancer treatment with graft preservation. The variability in immunosuppressive regimens across studies complicates the assessment of their role in UTUC progression. The limitations of the current evidence include small sample sizes, retrospective designs, and inconsistent follow-up durations. **Conclusions:** This SR underscores the need for tailored treatment strategies and improved long-term surveillance. Future research should focus on prospective studies with larger cohorts, exploring the impact of immunosuppression and novel therapies on UTUC outcomes in KTRs.

## 1. Introduction

**Upper urinary tract urothelial cell carcinoma (UTUC)** poses significant challenges for kidney transplant recipients (KTRs), largely due to the necessity of long-term immunosuppressive therapy [1]. Similarly to bladder carcinoma, UTUC is frequently diagnosed at an advanced stage, which complicates treatment and worsens prognosis. Patients often remain asymptomatic or presents with nonspecific symptoms, resulting in delayed detection and higher tumor grades at diagnosis compared to the immunocompetent population [2].

Treatment typically involves radical nephroureterectomy (RNU), which may be difficult to perform without compromising the function of the transplanted kidney. High-grade tumors add further complexity, and the frequent coexistence of bladder cancer (BC) introduces additional challenges [2]. Managing both UTUC and BC concurrently requires careful coordination to address multiple tumor sites, which may negatively affect treatment outcomes [3,4].

The ongoing need for immunosuppression further complicates the management of UTUC, as clinicians must balance the imperative of effective cancer treatment with the preservation of transplant function. This delicate balance impacts both the patient’s overall health and the long-term viability of the transplanted kidney.

Given these challenges, a thorough understanding of UTUC in KTRs is essential. Tailored treatment strategies are necessary to optimize patient outcomes and care. This systematic review (SR) aims to explore the unique challenges of UTUC in this high-risk population and provide insights for improving management practices.

## 2. Materials and Methods

### 2.1. Search Strategy

This systematic review was carried out in accordance with the PRISMA (Preferred Reporting Items for Systematic Reviews and Meta-Analyses) guidelines [5]. The protocol was submitted and recorded in the PROSPERO database (International Prospective Register of Systematic Reviews) under the registration number CRD42024592894. For this study, PubMed, Embase, Scopus, Web of Science, and Cochrane databases were investigated throughout March 2024 for UTUC in KTRs. The search strategies for each database are reported in the Supplementary Materials.

Article screening was realized by two authors (A.P. and B.L.) and any discrepancies were resolved through consultation with a third reviewer (A.T.). The primary aims of this SR were to evaluate the incidence and the clinical presentation of UTUC in patients previously submitted to kidney transplantation (KT), whilst the secondary aims of this SR were to report the surgical treatment approach and the oncological outcomes.

### 2.2. Study Selection

The study eligibility criteria were defined using the PICOS framework, which considers the population (P), intervention (I), comparator (C), outcome (O), and study design (S). For this review, the PICOS model was structured as follows:

- (P): adults (age > 18 yrs) previously submitted to KT, diagnosed with de novo UTUC after KT;
- (I): radical nephroureterectomy or endoscopic treatment
- (C): either comparative or noncomparative studies;
- (O): overall survival, cancer-specific survival;
- (S): prospective or retrospective studies.

**Figure 1:**
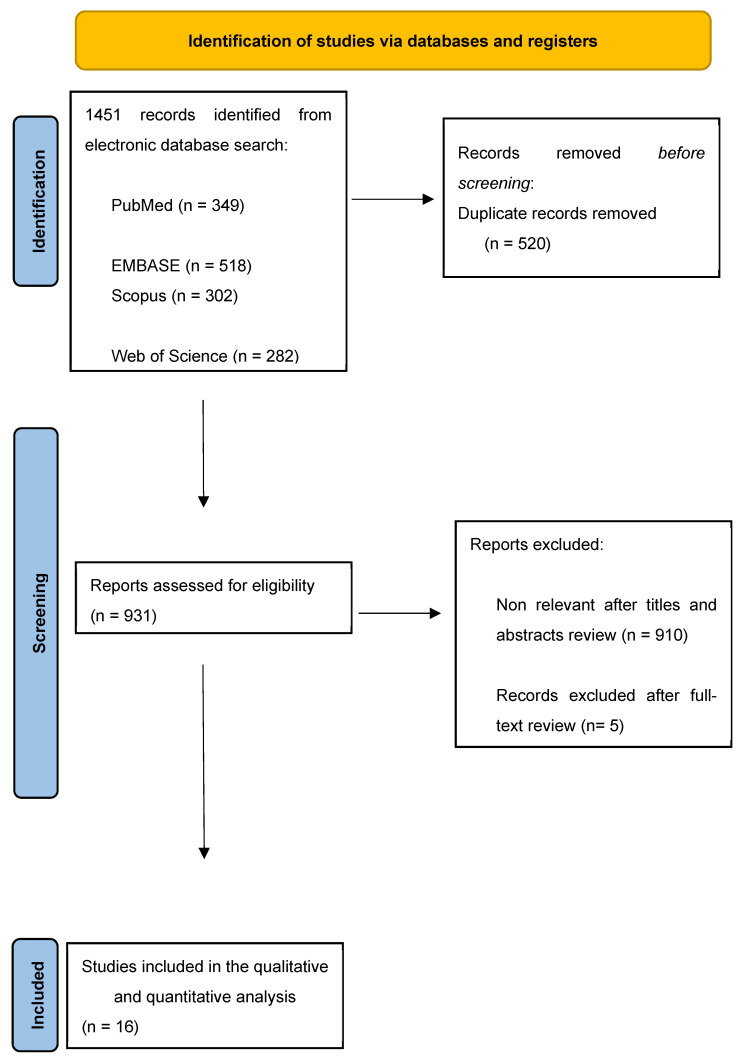
PRISMA flow chart—study selection with inclusion and exclusion criteria of the reviewed studies.

All studies containing relevant data were selected; only those with full-text availability in English and aligned with the PICO framework were included. The exclusion criteria comprised abstracts, editorials, commentaries, reviews, book chapters, non-English publications, and studies involving animal or cadaveric models (Figure 1).

### 2.3. Risk-of-Bias Assessment

**Figure 2:**
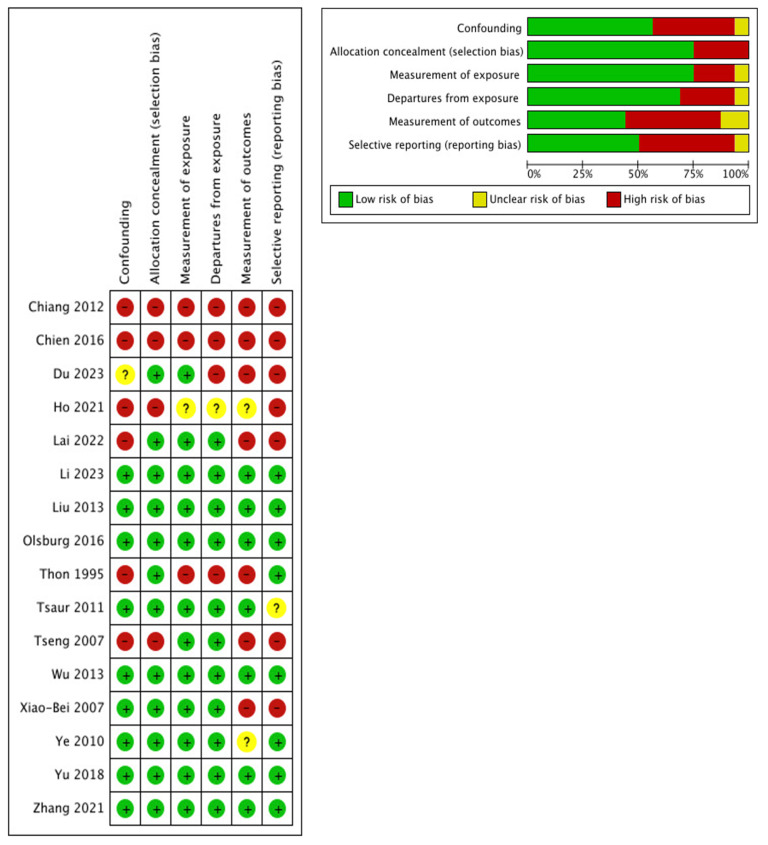
Evaluation of the risk-of-bias and confounders [2,6,7,8,9,10,11,12,13,14,15,16,17,18,19,20].

Two authors (A.P. and M.D.D.) independently conducted the risk of bias assessment using the ROBINS-I tool for non-randomized studies. Any disagreement was resolved through consultation with a third author (A.T.) (Figure 2).

### 2.4. Data Extraction and Analysis

Two authors (A.P. and B.L.) independently screened the articles based on predefined inclusion and exclusion criteria. Titles and abstracts were initially assessed, followed by a full-text review to verify eligibility for inclusion. Additionally, the reference lists of the selected studies were examined to identify any further relevant sources. Any disagreements regarding study selection were resolved by consulting a third reviewer (A.T.) (Figure 1).

## 3. Evidence Synthesis

### 3.1. Risk of Bias and Confounding Assessment

The risk of bias for the included studies is reported in Figure 2.

### 3.2. Study Characteristics

The literature search (Supplementary Materials) included 1451 records. After screening and eligibility assessment, 16 retrospective studies met the inclusion criteria (Table 1), comprising 526 patients diagnosed with UTUC. The studies reported a predominance of female patients, with percentages ranging from 50% (Tsaur et al. [18]) to 91.6% (Zhang et al. [12]). Male patients accounted for 7.7% to 50% of the cohorts. The median age at UTUC diagnosis varied across studies, ranging from 45 years (Olsburgh et al. [15]) to 61 years (Tsaur et al. [18]). Patients with a previous history of malignancies were generally excluded except for 2/16 studies, where patient with previous bladder cancer were included [6]. The mean time from KT to UTUC diagnosis also showed wide variability, with the shortest mean time of 7 months in Zhang et al. [12] and the longest of 181.3 months in Yu et al. [13] In the presence of suspicious findings on ultrasound, the diagnosis of UTUC in both the native upper urinary tract and the graft was established through computed tomography (CT) imaging. In cases with inconclusive results, diagnostic ureteroscopy with or without biopsy, as well as cystoscopy, was employed. Urinary cytology was reported in 3/16 studies [6,7,8].

### 3.3. Tumor Characteristics

Synchronous bilateral UTUC was uncommon, and was observed in 1% [13] to 16.6% [12] of patients. The tumor stage at diagnosis exhibited considerable heterogeneity across studies. Non-invasive Ta tumors were reported in 2.1% [2] to 29.2% [12] of patients, while the more invasive T3 and T4 stages occurred in 9.3% to 58.3% of cases [6]. The presence of carcinoma in situ (Tis) was rarely correctly reported, ranging from 0% [2] to 67.2% [10]. High-grade tumors were the most prevalent, with the highest prevalence reported by Chien et al. (96%) [14] and Yu et al. (100%) [13].

The incidence of concomitant bladder cancer (BC) varied between 18.7% [12] and 33% [8], whilst the incidence of synchronous bilateral UTUC among kidney transplant recipients ranged from 5.15% in the cohort reported by Du et al. [2] to 20.8% in the study by Liu et al. [6]. The vast majority of the studies reported data on UTUC arising in native kidneys. Only two studies provided data on UTUC occurring in the transplanted kidney. In particular, Yu et al. reported 1 case out of 10 involving the transplanted kidney, while the study by Olsburgh was entirely focused on four cases of UTUC in the transplanted kidney. Notably, this study was among those reporting the fewest potential biases. All patients included in the Olsburgh study were male, and no concomitant cases of UTUC in native kidneys were observed.

### 3.4. Treatment Approaches

The predominant treatment for UTUC in KTRs was radical nephroureterectomy with bladder cuff excision, either performed alone or in combination with transurethral resection of the bladder (TURB) if a concomitant bladder tumor was present. In the study of Zhang et al. [12], unilateral radical nephroureterectomy was performed in 56.2% of patients, whereas simultaneous bilateral radical nephroureterectomy was required in 43.8%. Yu et al. [13] reported radical nephroureterectomy in all cases, including a graft nephrectomy for UTUC in a graft kidney. In their study on UTUC in renal graft, Olsburgh et al. [15] detailed multiple surgical interventions, including attempted transplant ureterectomy converted to transplant nephroureterectomy, transplant pyelovesicostomy, and partial cystectomy. 

### 3.5. Follow-Up and Survival Outcomes

Follow-up duration varied widely between studies, ranging from 16.9 months [7] to 148 months [8]. Several studies reported overall survival (OS) at various timepoints. Du et al. [2] reported 5-year OS rates of 88.2% for patients with stage ≤T1 disease, while those with stage ≥T2 showed a 5-year OS of 90.2%. Li et al. [8] reported 5-year OS at 66.1%, with a notable decline to 49.7% at 10 years. Zhang et al. [12] reported an end-of-follow-up OS of 86%, while other studies reported OS rates of 80% [20] and 90.9% [16], respectively, at their final follow-up points.

Progression free survival (PFS) was reported in fewer studies but showed substantial variation. Li et al. [8] reported a 5-year PFS of 50.9%, while Du et al. [2] noted a 5-year PFS of 90.2% for stage ≥T2 disease. Recurrence-free survival was less frequently reported; Zhang et al. [12] showed an end-of-follow-up RFS of 68.8%, while Yu et al. [13] reported a similar RFS of 50%. Wu et al. [16] reported a 90.9% RFS at the end of follow-up.

Bladder recurrence rates ranged from 18.8% [12] to 61.2% [11]. Local recurrence and metastasis were documented in several studies, with rates varying from 20% [13] to 50% [15]. Zhang et al. [12] reported a UTUC controlateral recurrence rate of 33%.

Death attributable to UTUC was reported in several studies. Ho [11] reported UTUC-related death in 61.2% of cases. Yu et al. [13] documented a 20% death rate directly related to UTUC.

## 4. Discussion

This SR provides an overview of UTUC in patients following KT, focusing on patient characteristics, tumor staging, treatment strategies, and outcomes. The results highlight the complexity and heterogeneity of UTUC in this population, underscoring the need for careful monitoring and personalized therapeutic approaches.

The predominance of female patients across the included studies is notable, with some cohorts reporting female proportions as high as 91.6% [12], which contrasts with the general population, where UTUC shows a male predominance [21]. This divergence may reflect specific risk factors related to post-transplant immunosuppressive therapy, hormonal influences, or genetic predispositions that warrant further investigation. In fact, several studies in the literature suggest that the immunosuppressive regimens used in transplant patients, particularly calcineurin inhibitors, may contribute to an increased risk of developing UTUC, potentially altering the usual gender distribution observed in the general population [22]. Unfortunately, one of the limitations identified across the studies included in this review is the paucity of data regarding patients’ lifestyle habits, most notably a history of tobacco use, which was reported in only 3/16 studies [9,13,14].

Additionally, the prevalence of UTUC is not uniform across the reported studies. Notably, in cohorts of Asian patients, the use of molecules such as aristolochic acid, used in Chinese herbal medicine, has significantly impacted the incidence of chronic renal failure, and, consequently, UTUC. This is particularly evident in the case series provided by Zhang et al. [12], Lai et al. [10], Nortier et al. [23], and Du et al. [2], which highlight the role of aristolochic acid exposure in shaping the epidemiology of UTUC within Asian populations. However, data presented across different studies are cumulative, making it difficult to analyze the specific outcomes and characteristics of this population in isolation. More recent studies have investigated the role of exposure to aristolochic acid in the development of UTUC and bladder urothelial tumor recurrence after radical nephroureterectomy among the Balkan population [24].

The time from KT to UTUC diagnosis varied widely, with a mean of 7 months reported by Zhang et al. [12] and up to 181.3 months in Yu et al. [13]. This variation suggests that while some patients develop UTUC relatively early post-transplant, others may remain at risk for an extended period. These findings align with prior studies indicating that UTUC can occur several years after KT, with a long-term surveillance strategy being crucial for early detection and management. Notably, the high incidence of synchronous bilateral UTUC observed by Zhang et al. [12] further complicates the clinical picture and may require more aggressive treatment strategies, including bilateral nephroureterectomy. Considering the immunosuppression in KT recipients, the role of ureteroscopy, which is currently under investigation in non-transplanted patients [25,26], is very limited.

The treatment of choice across studies was predominantly radical nephroureterectomy with bladder cuff excision, which remains the gold standard for high-grade and invasive UTUC. The studies reviewed did not specify the surgical technique employed; however, given the history of prior transplantation, it is reasonable to assume that an open approach was always adopted. If a concomitant bladder tumor was diagnosed, an endoscopic TURB was performed prior or during the radical nephroureterectomy. The relatively high proportion of high-grade tumors (up to 100% [13]) and advanced-stage disease (T3-T4 stages present in up to 58.3% of patients in Liu 2013 [6]) underscores the aggressive nature of UTUC in this population. The reasons for this finding are likely multifactorial, including delayed diagnosis due to the lack of specific symptoms and the potential masking effects of immunosuppressive therapy. Considering this aspect, although it seems reasonable safe for low risk disease in the general population [27], a conservative endoscopic approach should not be considered in KT population.

Outcomes varied significantly across studies, with 5-year OS rates ranging from 16.7% to 90.9%. Patients with low-stage disease (Ta/T1) generally had better outcomes, with 5-year OS rates of 88.2% [2], while those with more advanced stages (≥T2) experienced markedly lower survival rates, with Li et al. [9] reporting 5-year OS as low as 50%. These outcomes are consistent with previous research on UTUC [21,28,29], where stage at diagnosis remains a critical prognostic factor. Additionally, high bladder recurrence rates were found, ranging from 18.8% to 61.2%, which aligns with the known high risk of bladder cancer development in patients with UTUC [30]. In the context of a screening strategy tailored to KT recipients, it could be useful to explore the role of specific markers for UTUC in this patient population, such as DNA Methylation Urine Biomarkers Test. In fact, while these markers have been primarily studied in bladder cancer for the general population, recent research has also investigated their applicability in UTUC [31].

However, despite these findings, there are several limitations in the current evidence that should be acknowledged. First, the retrospective nature of most included studies introduces potential biases, particularly selection bias, as patients with more severe disease may have been preferentially included. Moreover, there was significant heterogeneity between studies regarding follow-up duration, tumor staging, and treatment approaches, making direct comparisons challenging. Only a few studies provided detailed long-term follow-up data, and some lacked important outcome metrics, such as PFS and cancer-specific survival (CSS), limiting the ability to fully assess the long-term efficacy of treatment strategies.

Additionally, the variation in immunosuppressive regimens used across studies was not consistently reported, preventing an assessment of their potential role in disease progression or recurrence. Given the potential impact of different immunosuppressive agents on tumor development, future research should focus on this aspect to better understand the relationship between immunosuppression and UTUC outcomes.

Ultimately, the limited sample sizes in several studies—especially those focusing on graft UTUC—restrict the applicability of the results to broader populations. To confirm these findings and establish standardized management guidelines for UTUC in post-transplant patients, larger prospective studies are warranted. Furthermore, the role of novel therapeutic approaches, such as immune checkpoint inhibitors and targeted therapies, remains to be fully explored in this unique population.

## 5. Conclusions

This SR underscores the complexity of managing UTUC in KTRs, highlighting the need for individualized treatment strategies and improved surveillance. Radical nephroureterectomy remains the cornerstone of treatment; however, variability in outcomes across studies suggests that a more nuanced approach may be needed to optimize patient care. Future studies should aim to address the current gaps in the literature, particularly through prospective multicenter trials that explore the interplay between immunosuppression, UTUC progression and emerging therapies.

## Figures and Tables

**Table 1:** Data extraction.

Study	Number of Patients	Sex, *n* (%)	Age at UTUC Diagnosis, Years	Time from KT to UTUC Diagnosis, Months, Mean (SD)	Synchronous Bilateral UTUC, *n* (%)	UTUC Pathological Stage (TNM Classification), *n* (%)	Concomitant BC	UTUC Grade, *n* (%)	Treatment	Median FU, Months (Range)	OS, Timepoint (%)	PFS (%)	RFS (%)	Bladder Recurrence, *n* (%)	Local Recurrences/Metastasis, *n* (%)	Death for UTUC, *n* (%)
Du 2023 [2]	97	F: 77 (79.4) M: 20 (20.6)	NA	98.1 (66.4)	5 (5.15)	Ta: 2 (2.1) Tis: 0 (0) T1: 25 (25.8) T2: 14 (14.4) T3: 9 (9.3) T4: 1 (1.03)	29 (29.9)	1: 15 (15.5) 2: 40 (41.2) 3: 25 (25.8)	Radical nephroureterectomy ± TURB	NA	UTUC Stage ≤ T1, 5 yrs 88.2 Stage ≥ T2, 5 yrs 90.2 UTUC + BC Stage ≤ T1, 5 yrs 57.7 Stage ≥ T2, 5 yrs 48.2	NA	NA	NA	NA	NA
Li 2023 [9]	106	F: 89 (83.9) M: 17 (16.0)	57 (51–62)	91.5 (48–143.75)	7 (6.6)	Ta: 6 (5.7) Tis: 0 (0) T1: 46 (43.4) T2: 17 (16) T3: 28 (26.4) T4: 9 (8.5)	33 (31.13)	1: 3 (2.83) 2: 53 (50) 3: 50 (47.2)	Radical nephroureterectomy ± TURB	96 (55–148) months	1 year: 88.3 5 yrs: 66.1 10 yrs: 49.7	NA	1 year: 80.4 3 yrs: 68.5 5 yrs: 50.9	24 (22.6)	35 (33.0) (controlateral recurrence)	41 (38.7)
Lai 2022 [10]	61	F: 38 (62.3) M: 23 (37.7)	NA	79.7 (NA)	NA	Renal pelvis: Ta: 6 (9.8) T1: 9 (14.8) T2–T4: 19 (31.1) Ureter tumor: Ta: 10 (16.4) Tis: 41 (67.2) T1: 9 (14.8) T2–T4: 18 (29.5)	NA	NA	Radical nephroureterectomy	58.8 (NA)	NA	NA	NA	NA	NA	NA
Ho 2021 [11]	67	F:49 (73.1) M:18 (26.9)	NA	7.53 (NA)	14 (15.9)	Ta/is: 7 (10.4) T1: 16 (23.9) T2: 13 (19.4) T3: 27 (40.3) T4: 0 (0)	NA	1: 4 (5.9) 2/3: 56 (83.6) Unknown: 7 (10.4)	NA	118.6 (70.2)	NA	NA	NA	41 (61.2)	NA	NA
Zhang 2021 [12]	48	F:44 (91.6) M:4 (8.3)	58.5 (52–64)	7 (4–10)	8 (16.6)	Ta/1: 14 (29.2) T2: 15 (31.2) T3: 13 (27.1) T4: 2 (4.2)	9 (18.7)	Low-grade: 5 (10.4) High-grade: 43 (89.6)	Unilateral radical nephroureterectomy: 27 (56.2) Simultaneous bilateral radical nephroureterectomy: 21 (43.8)	65 (33.8– 90.5)	End of FU: 86	NA	End of FU: 68.8	9 (18.8)	15 (33.3)	12 (25)
Yu 2018 [13]	10 (9 in native UT 1 in graft UT)	F: 8 (80) M: 1 (10)	45.9 (8.5)	181.3 (73)	1 (10)	T1: 2 (20) T2: 2 (20) T3: 5 (50) (1 in the graft) T4: 1 (10)	NA	High-grade: 10 (100)	Radical nephroureterectomy	71.8 (47.9)	End of FU: 80	End of FU: 50	End of FU: 50	3 (30)	Recurrence: 5 (50) Progression: 5 (50)	2 (20)
Chien 2016 [14]	25	F:17 (68) M:8 (32)	54.1 (5.6)	NA	NA	Ta/Tis/T0: 5 (20) T1: 6 (24) T2: 7 (28) T3: 7 (28) T4: 0 (0)	12 (48)	Low-grade: 1 (4) High-grade: 24 (96)	NA	NA	NA	NA	NA	12 (48)	6 (24)	NA
Olsburgh 2016 [15]	4 (graft tumor)	M: 4 (100%)	45 (14.4)	21.8 (9.4)	0 on native UTs	T1: 1 (25) T2: 1 (25) T3: 2 (50)	0 (0)	3: 2 (50) 3 + cis: 2 (50)	- Attempted transplant Ureterectomy but converted to TNU - Transplant ureterectomy + Pyelovesicostomy - Initially ureteric resection + pyelovesicostomy - Transplant pyelo-ureterectomy + partial cystectomy. Subsequent transplant pyelovesicostomy and excision of right native ureter	34 (21)	End of FU: 50	End of FU: 50	End of FU: 50	NA	2 (50)	2 (50)
Liu 2013 [6]	24	F:14 (58.3) M:10 (41.6)	53.5 (9)	NA	5 (20.8)	Ta/1: 4 (16.7) T2: 6 (25) T3/4: 14 (58.3)	13 (54.2)	NA	Radical nephroureterectomy	31 (range 4–94)	End of FU: 80	End of FU: 33.3	NA	7 (29.2)	8 (33.3)	4 (16.7)
Wu 2013 [16]	11	F:10 (90.9) M:1 (9.09)	54–74	38.7 ± 16.0	2 (18.2)	T1: 5 (45.4) T2: 5 (45.5) T2 + N1: 1 T3: 2	1 (9.1)	NA	Radical nephroureterectomy	21.7 (3–48)	End of FU: 90.9	End of FU:90.9	NA	2 (18.2)	1 (9.1)	1 (9.1)
Chiang 2012 [17]	45	F:22 (48.9) M:23 (51.1)	NA	67.4	NA	NA	22 (48.9)	LG: 1 (2.2) HG: 44 (97.8)	Radical nephroureterectomy	NA	End of FU: 26.7	NA	NA	NA	NA	12 (26.7)
Tsaur 2011 [18]	6	F:3 (50) M:3 (50)	61.0 (6.6)	66.0 (70.7)	0 (0)	Ta/1: 0 (0) T2: 1 (16.7) T3: 4 (66.7) T4: 1 (16.7)	4 (66.7)	G1: 0 (0) G2: 1 (16.7) G3: 5 (83.3)	Radical nephroureterectomy +/- TURBT	115 (range NA)	End of FU: 66.7	End of FU: 66.6	NA	NA	2 (33.3)	1 (16.7)
Ye 2010 [19]	13	F: 12 (92,3%) M: 1 (7.69%)	56.3 (9.4)	51.3 (32.6)	2 (15.4)	T1: 7 (53.8) T1+ is: 1 (7.7) T2: 5 (38.5)	4 (30.8)	1: 1 (7.7) 2: 6 (46.1) 3: 9 (69.2)	Radical nephroureterectomy + TURBT + chemotherapy	30 (10–43)	100	NA	NA	2 (15.4)	2 (15.4)	0 (0)
Tseng 2007 [20]	9	F: 6 (66.67) M: 3 (33.33)	48.3 (range 41–63)	11–99.6	NA	Ta: 2 (22.2) Tis/T1–T4: 7 (77.7)	NA	NA	transvesical ureterectomy + radical nephroureterectomy	NA	NA	NA	NA	NA	1 (11.1)	NA
Li 2008 [8]	11	F: 9 (81.8) M: 3 (27.3)	57.3 years (range 44–76)	44.1 (range 16–89)	1 (9.1)	Ta: 1 (9.1) T1: 9 (81.8) T2: 1 (9.1)	2 (18.2)	G1: 1 (9.1) G2: 9 (81.8) G3: 1 (9.1)	Radical nephroureterectomy ± TURB	26 (8–65).	End of FU: 81.8	NA	NA	NA	1 (9.1)	3 (27.3)
Thon 1995 [7]	6	NA	56.1 (51–66)	72 (42–108)	1 (16.7)	Tis: 1 (16.7) Ta: 2 (33.3) T1: 1 (16.7) T2: 1 (16.7) T3: 1 (16.7)	2 (2)	G1: 4 (66.7) G2: 1 (16.7)	Nephroureterectomy ± TURB	16.9 (4–33)	End of FU: 16.7	End of FU: 83.3	NA	NA	NA	3

Abbreviations. N: number; F: female; M: male; BC: bladder cancer; UT: upper tracts; UTUC: upper tract urothelial cancer; TURB: transurethral resection of the bladder; SD: standard deviation; TNU: transplant nephroureterectomy; FU: follow up; OS: overall survival; PFS: progression-free survival; yrs: years; UT: upper urinary tract.

## Supplementary Materials

The following supporting information can be downloaded at: https://www.mdpi.com/article/10.3390/jcm14113927/s1, File S1: Search strings.
