# Supplementary material for: Systematic Review on Upper Urinary Tract Carcinoma in Kidney Transplant Recipients
Source: J Clin Med. 2025 Jun 3;14(11):3927. doi: 10.3390/jcm14113927 (PMC12155726; doi:10.3390/jcm14113927)
Supplement: Supplementary file 1 [file jcm-14-03927-s001.zip › jcm-3553288-supplementary.pdf]

Supplementary matherials: search strings.

OVID Medline

1 exp Kidney Transplantation/
2 ((kidney* OR renal) ADJ3 (transplant* OR graft* OR homotransplant* OR allotransplant* OR allograft* OR homograft*)).ti,ab,kf.
3 (post-KT).ti,ab,kf.
4 1 OR 2 OR 3
5 exp Urothelium/ AND exp Carcinoma/
6 exp Carcinoma, Transitional Cell/
7 ((urothel* OR upper tract OR upper urinary tract) ADJ3 (carcino* OR cancer* OR neoplas* OR tumo?r* OR malignan* OR metasta*)).ti,ab,kf.
8 (UTUC OR UT-UC OR UTUCs OR UT-UCs).ti,ab,kf.
9 (transitional cell carcinoma* OR metastatic transitional cell*).ti,ab,kf.
10 5 OR 6 OR 7 OR 8 OR 9
11 4 and 10

Embase

#1 'kidney transplantation'/exp
#2 ((kidney* OR renal) NEAR/3 (transplant* OR graft* OR homotransplant* OR allotransplant* OR allograft* OR homograft*)):ti,ab,kw
#3 post-KT:ti,ab,kw
#4 #1 OR #2 OR #3
#5 'urothelium'/exp AND 'carcinoma'/exp
#6 'urothelial tumor'/exp
#7 ((urothel* OR 'upper tract' OR 'upper urinary tract') NEAR/3 (carcino* OR cancer* OR neoplas* OR tumo$r* OR malignan* OR metasta*)):ti,ab,kw
#8 (UTUC OR UT-UC OR UTUCs OR UT-UCs):ti,ab,kw
#9 ('transitional cell carcinoma*' OR 'metastatic transitional cell*'):ti,ab,kw
#10 #5 OR #6 OR #7 OR #8 OR #9
#11 #4 AND #10

Cochrane CENTRAL

#1 MeSH descriptor: [Kidney Transplantation] explode all trees
#2 ((kidney* OR renal) NEAR/3 (transplant* OR graft* OR homotransplant* OR allotransplant* OR allograft* OR homograft*)):ti,ab,kw
#3 (post-KT):ti,ab,kw
#4 #1 OR #2 OR #3
#5 MeSH descriptor: [Urothelium] explode all trees
#6 MeSH descriptor: [Carcinoma] explode all trees
#7 #5 AND #6
#8 MeSH descriptor: [Carcinoma, Transitional Cell] explode all trees

#9 ((urothel* OR 'upper tract' OR 'upper urinary tract') NEAR/3 (carcino* OR cancer* OR neoplas* OR tumo?r* OR malignan* OR metasta*)):ti,ab,kw
#10 (UTUC OR UT-UC OR UTUCs OR UT-UCs):ti,ab,kw
#11 ((transitional NEXT cell NEXT carcinoma*) OR (metastatic NEXT transitional NEXT cell*)):ti,ab,kw
#12 #7 OR #8 OR #9 OR #10 OR #11
#13 #4 AND #12

Scopus

#1 TITLE-ABS-KEY((kidney* OR renal) W/2 (transplant* OR graft* OR homotransplant* OR allotransplant* OR allograft* OR homograft*))
#2 TITLE-ABS-KEY(post-KT)
#3 #1 OR #2
#4 TITLE-ABS-KEY((urothel* OR "upper tract" OR "upper urinary tract") W/2 (carcino* OR cancer* OR neoplas* OR tumor* OR tumour* OR malignan* OR metasta*))
#5 TITLE-ABS-KEY(UTUC OR UT-UC OR UTUCs OR UT-UCs)
#6 TITLE-ABS-KEY("transitional cell carcinoma*" OR "metastatic transitional cell*")
#7 #4 OR #5 OR #6
#8 #3 AND #7

Web of Science

#1 TS=((kidney* OR renal) NEAR/2 (transplant* OR graft* OR homotransplant* OR allotransplant* OR allograft* OR homograft*))
#2 TS=(post-KT)
#3 #1 OR #2
#4 TS=((urothel* OR "upper tract" OR "upper urinary tract") NEAR/2 (carcino* OR cancer* OR neoplas* OR tumo$r* OR malignan* OR metasta*))
#5 TS=(UTUC OR UT-UC OR UTUCs OR UT-UCs)
#6 TS=("transitional cell carcinoma*" OR "metastatic transitional cell*")
#7 #4 OR #5 OR #6
#8 #3 AND #7
